# Supplementary material for: The interactome of intact mitochondria by cross-linking mass spectrometry provides evidence for coexisting respiratory supercomplexes
Source: Mol Cell Proteomics. 2017 Dec 8;17(2):216–32. doi: 10.1074/mcp.RA117.000470 (PMC5795388; doi:10.1074/mcp.RA117.000470)
Supplement: Supplemental Data [file supp_RA117.000470_133922_0_supp_23978_fzffwf.zip › spectra_annotation/mito_DR_spectra_annotation/413-1-4-1-8-1.pdf]

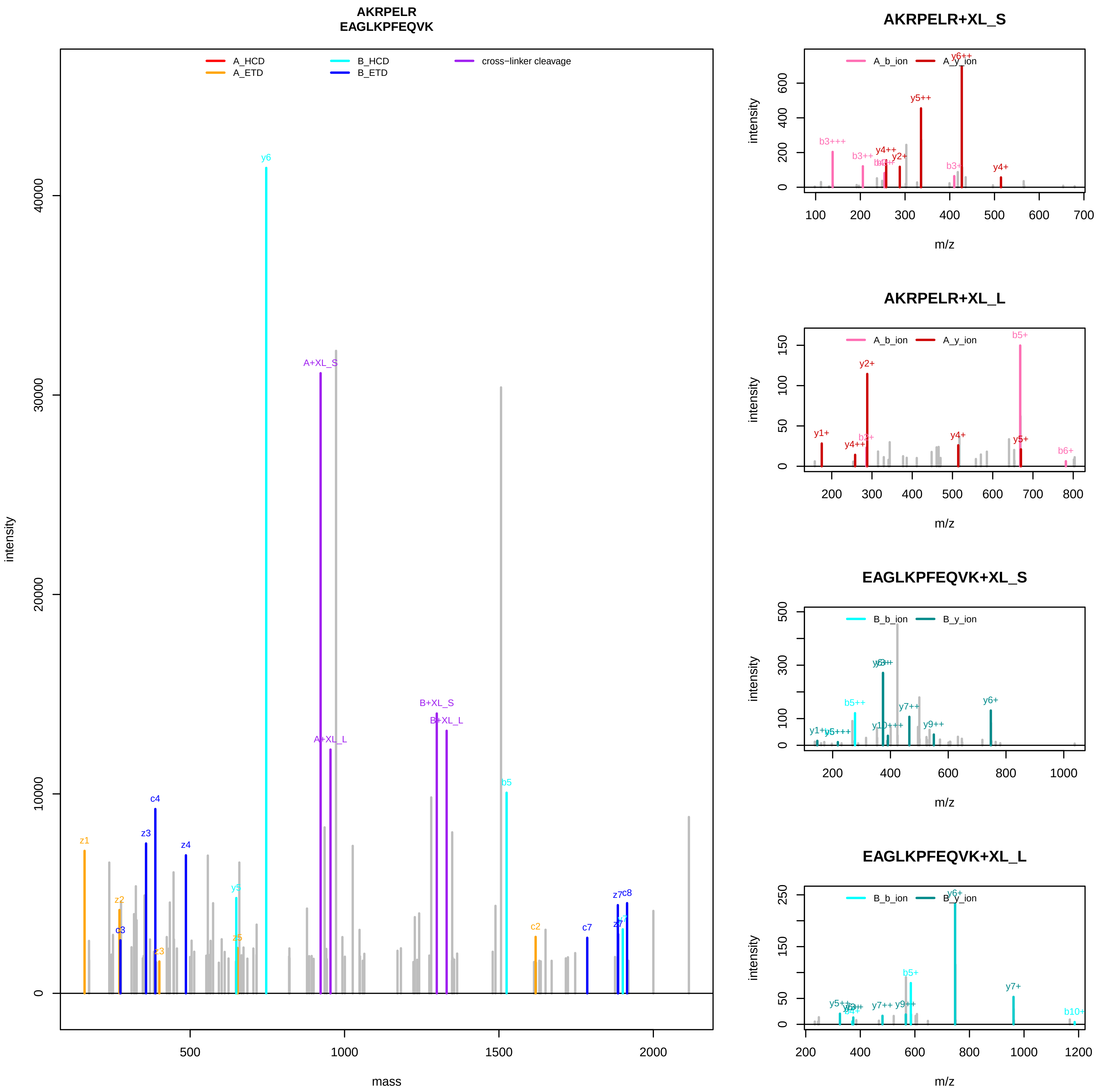

AKRPELR
EAGLKPFEQVK
A_HCD
A_ETD
B_HCD
B_ETD
cross−linker cleavage
intensity
mass
0
10000
20000
30000
40000
500
1000
1500
2000
y6
A+XL_S
A+XL_L
B+XL_S
B+XL_L
b5
z1
z2
c3
z3
c4
z4
y5
z5
c2
c7
z7
c8
AKRPELR+XL_S
A_b_ion
A_y_ion
b3+++
b3++
y4++
y2+
y5++
y6++
b3+
y4+
m/z
AKRPELR+XL_L
y1+
y4++
b2+
y2+
y4+
y5+
b5+
b6+
EAGLKPFEQVK+XL_S
B_b_ion
B_y_ion
y1+
b5++
y7++
y9++
y6+
EAGLKPFEQVK+XL_L
y5++
y7++
y9++
b5+
y6+
y7+
b10+
